# Progress of Immunotherapies in Parkinson's Disease

**DOI:** 10.1002/cns.70964

**Published:** 2026-06-02

**Authors:** Yong Peng, Xu‐hui Kang, Shun‐yu Yao, Xiuli Zhang, Sugimoto Kazuo, Jia Liu, Miao‐qiao Du, Lan‐xin Lin, Dai‐yi Jiang, Quan Chen, Hong Jin

**Affiliations:** ^1^ Department of Neurology First Affiliated Hospital of Hunan Traditional Chinese Medical College (Hunan Provincial Directly Affiliated Hospital of Traditional Chinese Medicine) Zhuzhou China; ^2^ Department of Neurology Provincial Hospital of Traditional Chinese Medicine Affiliated to Hunan University of Traditional Chinese Medicine Zhuzhou China; ^3^ Science and Technology Innovation Center Hunan University of Chinese Medicine Changsha China; ^4^ Department of Neurology Dongzhimen Hospital, Beijing University of Chinese Medicine Beijing China; ^5^ Institute for Brain Disorders Beijing University of Chinese Medicine Beijing China

**Keywords:** immune systems, immunotherapy, Parkinson's disease

## Abstract

**Background:**

Immune dysregulation plays a pivotal role in the pathogenesis and progression of Parkinson's disease (PD). Increasing evidence suggests that immunotherapies targeting α‐synuclein (α‐syn) pathology may offer potential disease‐modifying strategies for PD.

**Objective:**

This review aims to systematically summarize recent advances in α‐syn–targeted immunotherapies for PD and to evaluate the translational challenges and future directions of precision immunotherapy.

**Methods:**

We critically reviewed the preclinical evidence and clinical trial outcomes of both active immunization strategies, including PD01A and UB‐312, and passive monoclonal antibody therapies, such as prasinezumab and cinpanemab.

**Results:**

Early‐phase studies demonstrated favorable safety profiles and robust peripheral target engagement for several immunotherapeutic candidates. However, recent Phase II clinical trials, including PASADENA and SPARK, failed to achieve their primary clinical endpoints, highlighting a substantial gap between biological target engagement and meaningful clinical benefit. Emerging strategies aimed at overcoming these limitations include stage‐specific interventions, optimized patient stratification, and differentiated therapeutic approaches.

**Conclusion:**

Although current α‐syn–targeted immunotherapies have shown limited clinical efficacy, ongoing advances in precision immunotherapy and individualized intervention strategies may help overcome existing therapeutic bottlenecks. This review provides a strategic framework for the future development of disease‐modifying therapies for PD.

## Graphical abstract

Parkinson's disease (PD) is caused by an immune disorder triggered by α‐synuclein, leading to a persistent neuroinflammatory cycle that drives the disease from non‐motor symptoms to the motor stage. Second, it presents active and passive immunotherapy strategies targeting α‐synuclein and their potential in slowing down the disease progression. At the same time, it emphasizes the challenges and future prospects of clinical trials.

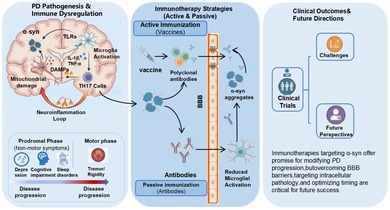

**Abbreviations**
BBB: blood–brain barrier
CNS: central nervous system
CSF: cerebrospinal fluid
DA: dopamine agonists
DAMPs: damage‐associated molecular
ENS: enteric nervous system
FcRγ: Fcγ receptor
HIF‐1a: hypoxia‐inducible factor‐1a
ICAM‐1: intercellular adhesion molecule 1
IIM: innate immune memory
IL‐17: interleukin 17
IL‐1β: interleukin‐1β
LFA‐1: function‐associated antigen 1
MAO‐B: monoamine oxidase‐B
MS: multiple sclerosis
NF‐kB: the nuclear factor‐kB
OB: olfactory bulb
OD: olfactory dysfunction
OH: orthostatic hypotension
PD: Parkinson's disease
PDD: dementia
PD‐MCI: mild cognitive impairment
SCD: subjective cognitive decline
SCFAs: short‐chain fatty acids
TLR2: Toll‐like receptor 2
TLRs: Toll‐like receptors
α‐syn: alpha‐synuclein

## 1 Introduction

Parkinson's disease (PD) ranks as the second most prevalent neurodegenerative condition and progresses over time. Globally, approximately 5–35 people per 100,000 are newly diagnosed with PD annually. Among the older population aged ≥ 65 years, the prevalence ranges 1%–2% [1]. The total number of patients with PD is predicted to double by 2030 compared to 2005 [1, 2]. In recent years, the average age of onset for patients with PD has shown a significant downward trend, and the analysis based on gender distribution shows that the risk of men contracting this disease is higher than that of women [3]. In clinical practice, the main features of PD are a series of motor disorders, such as slow movements, tremor at rest, muscle rigidity, and changes in posture and walking patterns. As the disease progresses, patients gradually develop progressive functional impairments, which affect the daily living ability of patients with PD and have a significant impact on their quality of life [4]. These alterations in motor symptoms are strongly associated with the degenerative modifications in the substantia nigra and the reduced dopamine levels in the striatum [5]. Nonmotor symptoms encompass a range of issues such as diminished olfactory function, gastrointestinal disturbances such as constipation, mood disorders including depression, various sleep disturbances, cognitive impairments such as memory issues, urinary dysfunction, orthostatic hypotension, and sensations of pain, among others. and they may be associated with neurodegeneration in other structures such as the peripheral autonomic nervous system [6, 7]. This will significantly affect the quality of life of patients and impose higher demands on the nursing staff. Despite the availability of symptomatic treatment options for PD, ultimately, patients will encounter cumulative disability and loss of independence as a consequence of the progression of PD [8].

**FIGURE 1:**
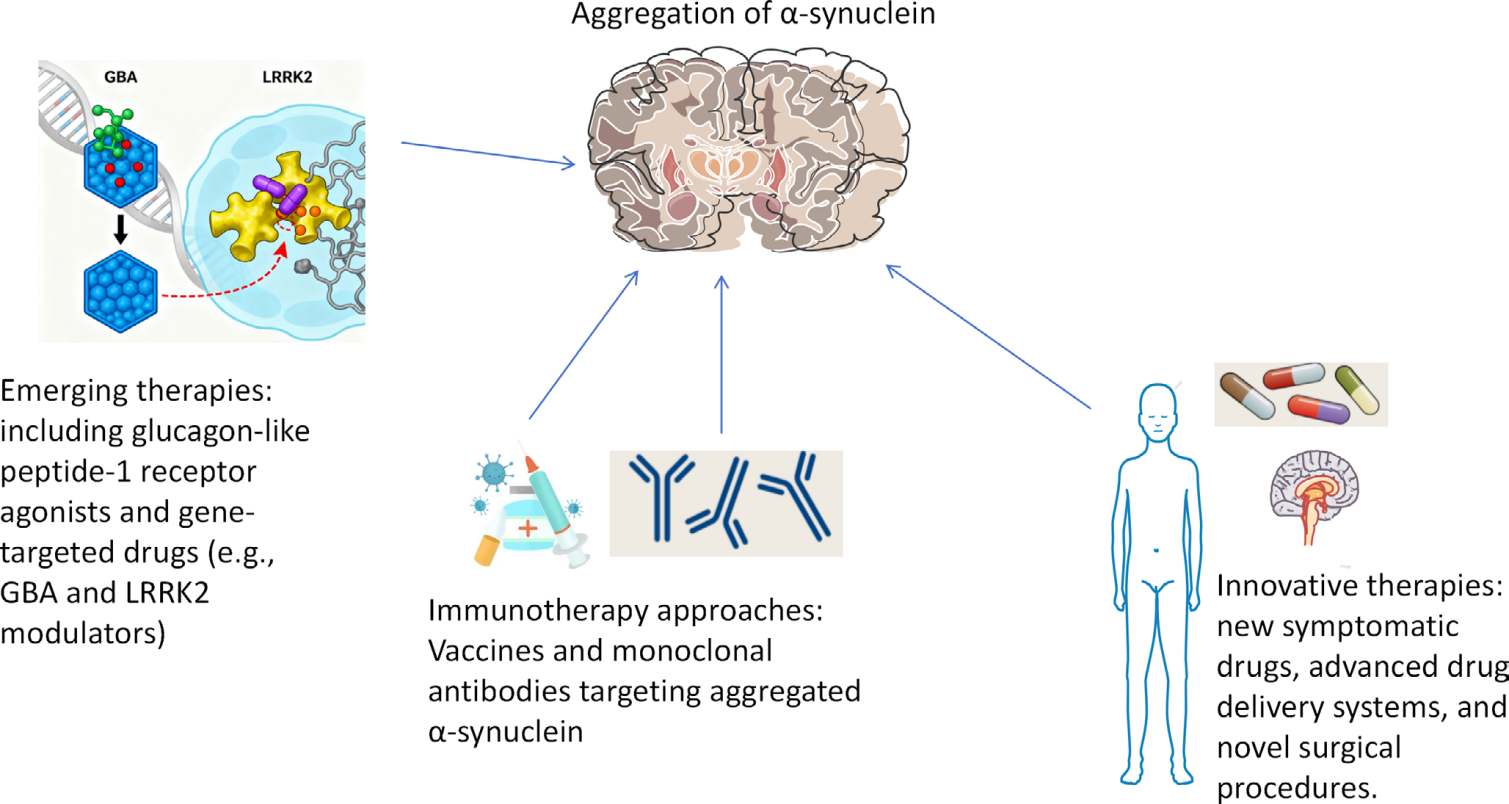
Advances in Parkinson's disease (PD) treatments. This figure highlights recent progress in the treatment strategies for PD, centered on the hallmark pathology of α‐syn aggregation. It systematically presents: immunotherapies, including vaccines and monoclonal antibodies targeting aggregates to block toxic effects; emerging disease‐modifying therapies, such as GLP‐1 receptor agonists and gene‐targeted agents (GBA and LRRK2 modulators); and innovative interventions to improve patient prognosis, including novel symptomatic medications, advanced drug delivery systems, and cutting‐edge surgical techniques. α‐syn, alpha‐synuclein; GLP‐1 glucagon‐like peptide‐1; GBA, glucocerebrosidase alpha; LRRK2, leucine‐rich repeat kinase 2.

One of the significant hindrances in the advancement of potential treatments for brain protection lies in the deficiency of reliable and sensitive markers for disease progression. Currently, clinical trials are exploring immunotherapies, such as vaccines or monoclonal antibodies targeting aggregated and harmful α‐syn, as well as approaches aimed at preventing protein aggregation or enhancing protein clearance. The guarded optimism arises from the exploration of glucagon‐like peptide one receptor agonists, specific agents targeting genes associated with PD (such as GBA or LRRK2 modifiers), and other promising drugs that have the potential to modify the course of the disease. Emerging treatments, such as new symptomatic drugs, innovative drug delivery systems, and novel surgical interferences, bestow hope upon patients with PD with regard to their future outcomes and prognosis [9]. Immunotherapy is a current research hotspot in PD, and plenty of animal experiments and clinical trials have shown the expected effect [10] (see Figure 1).

This review aims to provide a comprehensive overview of the current landscape of immunotherapy in PD, with a particular emphasis on distinguishing between active and passive immunization strategies and evaluating their key outcomes. Our primary contribution lies in systematically comparing the mechanisms of action, clinical trial results, and associated challenges of these two approaches, thereby offering a clear framework for understanding their respective potentials and limitations. Furthermore, we explore the critical role of early nonmotor symptom identification in the context of disease management and immunotherapeutic intervention. Finally, we summarize the pipeline of novel agents currently under clinical investigation.

The article is structured as follows: after introduction, we delineate the immunopathological basis of PD. We then critically assess the current evidence for active and passive immunotherapies. Subsequent sections discuss the management of nonmotor symptoms and review emerging drugs in clinical trials, concluding with future perspectives on the field.

Early detection of PD, particularly through its nonmotor symptoms, plays a crucial role in disease management. These nonmotor symptoms often appear 10–20 years before the onset of typical motor disorders, providing a valuable window for early intervention and thereby improving patient prognosis [11, 12]. Nonmotor symptoms of PD are closely linked to immune‐mediated α‐syn pathology and neuroinflammation, serving as a key avenue for understanding disease origins and identifying early diagnostic biomarkers.

## 2 Brief Introduction of Nonmotor Symptoms of PD

The most prominent nonmotor symptom of PD is olfactory dysfunction (OD), which affects up to 72% of patients [13, 14]. Within 5 years of a PD diagnosis, up to 80% of patients will experience sleep disorders, although many do not report these concerns to their doctors [15, 16, 17, 18]. Cognitive impairment may occur several years to decades before a formal diagnosis, with considerable interindividual variability [19]. Evidence suggests that α‐syn initially aggregates in the olfactory system [20]. Furthermore, the olfactory bulb (OB) may be one of the earliest affected regions [21]. The formation of Lewy bodies composed of misfolded α‐syn within the OB is a characteristic of the early stage of PD [22]. Additionally, in structural magnetic resonance imaging, OD is associated with degeneration of the basal forebrain cholinergic pathway, bilateral insular cortex, amygdala, and hippocampus [23, 24]. The formation of Lewy bodies in the OB suggests that similar pathological changes may occur in other brain regions [21, 25]. Whether OD is related to the duration and severity of the disease remains unclear [26, 27]. Abnormal aggregation of α‐syn affects the medulla oblongata and pons before the onset of motor symptoms, damaging key structures that regulate sleep and leading to sleep problems [28, 29]. The decline in plasma dopamine levels at night is part of the reason for sleep disorders [30, 31, 32], but the specific mechanism is not yet clear [33].

Cognitive impairment includes a spectrum of conditions, from subjective cognitive decline (SCD) to mild cognitive impairment (PD‐MCI), and ultimately to dementia (PDD) [34, 35]. Approximately one‐third of the patients met the criteria for mild cognitive impairment at the time of diagnosis, and most of them will eventually develop dementia [36, 37, 38]. Studies have shown that in the progression of PD‐MCI and PDD, the innervation of vasopressin fibers in the basal forebrain cholinergic neurons increases, which may be a response to the increase in α‐syn positive cells [39]. In recent years, nonpharmacological therapies have received more attention in the treatment of cognitive impairment [40].

Constipation symptoms may exist for up to 20 years before motor symptoms appear in PD and may affect approximately 20%–29% of PD patients [41, 42]. Aggregation of α‐syn can be detected early in the stomach and intestines, [43] possibly first occurring in the enteric nervous system and then spreading to brain regions through the vagus nerve [44]. The colon of constipation patients shows a decreased immune response to dopamine and an increased expression of D1 receptor mRNA [45, 46]. The latest research indicates that α‐syn pathology is an important factor in gastrointestinal motility disorders, [47] and short‐chain fatty acids can promote α‐syn–mediated neuroinflammation [48, 49]. Depression is one of the main causes of disability worldwide [50]. Patients with PD are more prone to depression than normal people [51]. Approximately 20%–30% of patients with PD have depressive symptoms, and these mood disorders sometimes occur before motor symptoms [52]. Due to overlapping clinical manifestations, accurately diagnosing the depressive state of patients with PD is challenging [53]. Research indicates that the depressive‐like symptoms observed in patients with PD can be attributed to neurodegenerative changes, involving neurotransmitter imbalances in serotonergic, noradrenergic, and cholinergic nuclei [54]. A recent study shows that stress can interfere with gut microbiota, increasing pro‐inflammatory cytokine levels, and worsening depressive symptoms in PD patients [53].

## 3 PD and the Immune System

### 3.1 The Key Role of α‐Syn in the Pathogenesis of PD

The abnormal aggregation of α‐syn within neurons constitutes a core pathological hallmark of synucleinopathies such as PD. This process not only directly impairs neuronal function but also initiates a vicious cycle of “protein aggregation–mitochondrial damage–inflammatory activation” by triggering mitochondrial dysfunction and the release of mitochondrial‐derived damage‐associated molecular patterns (DAMPs), which subsequently activate microglia‐mediated neuroinflammation. Mitochondrial DAMPs are recognized by microglia, leading to amplified inflammatory responses via pathways such as cGAS–stimulator of interferon genes (STING), TLR9, and P2X7. Concurrently, inflammatory cytokines exacerbate mitochondrial damage in neurons. Sustained inflammation and mitochondrial impairment ultimately result in extensive neuronal death, and the subsequent release of additional α‐syn aggregates and DAMPs from dying neurons perpetuates microglial activation, thereby driving the pathological progression of PD and related disorders [55].

By constructing in vivo and in vitro models of PD induced by α‐syn prefibrillar assemblies (α‐syn PFFs), the molecular mechanism of oxidative damage to dopaminergic neurons mediated by exogenous α‐syn PFFs through the “p‐NMDAR2B/Nur77” pathway was revealed for the first time. Exposure to exogenous α‐syn PFFs activates Fyn kinase, phosphorylates the Tyr1472 site of NMDAR2B (increasing p‐NMDAR2B), inhibits Nur77 expression, intensifies oxidative stress, causes mitochondrial damage, and ultimately leads to the death of dopaminergic neurons [56].

Significant iron overload and abnormal aggregation of α‐syn have been observed in the substantia nigra of PD. Moreover, abnormal function of Parkin (a familial PD‐related E3 ubiquitin ligase) is closely related to sporadic PD. Iron overload causes oxidative inactivation of Parkin (Parkin contains 35 cysteines and is easily oxidized and inactivated by ROS generated by iron catalysis), and the ubiquitination and degradation of α‐syn are blocked, leading to abnormal accumulation. This promotes the targeting of α‐syn to mitochondria, induces opening of mPTP and ATP production, and ultimately causes the death of dopaminergic neurons [57].

α‐Syn aggregation mediates ferroptosis of dopaminergic neurons through the “iron‐dependent lipid peroxidation–membrane interaction–calcium disorder” pathway [58]. α‐Syn aggregation mediates ferroptosis of dopaminergic neurons through the “iron‐dependent lipid peroxidation–membrane interaction–calcium disorder” pathway. α‐Syn becomes the “final executor molecule” for erastin‐induced cell death by binding to mitochondria and activating the mPTP [59].

The regulatory role of α‐syn in ferroptosis of dopaminergic neurons in PD was first revealed. It was found that α‐syn affects neuronal sensitivity to ferroptosis by regulating the composition of ether phospholipids (ether‐PLs) in the cell membrane. Low expression of α‐syn protects neurons, whereas high expression exacerbates ferroptosis. By maintaining ether‐PL content, α‐syn provides “substrates” for lipid peroxidation related to ferroptosis. Abnormal α‐syn levels, o either low or high, alter ether‐PL composition, thereby affecting neuronal sensitivity to ferroptosis. This study established a direct link between α‐syn and ferroptosis for the first time, highlighting ether phospholipids as key intermediary and improving the pathological chain of “α‐syn aggregation–oxidative stress–neuronal death” in PD [60].

**FIGURE 2:**
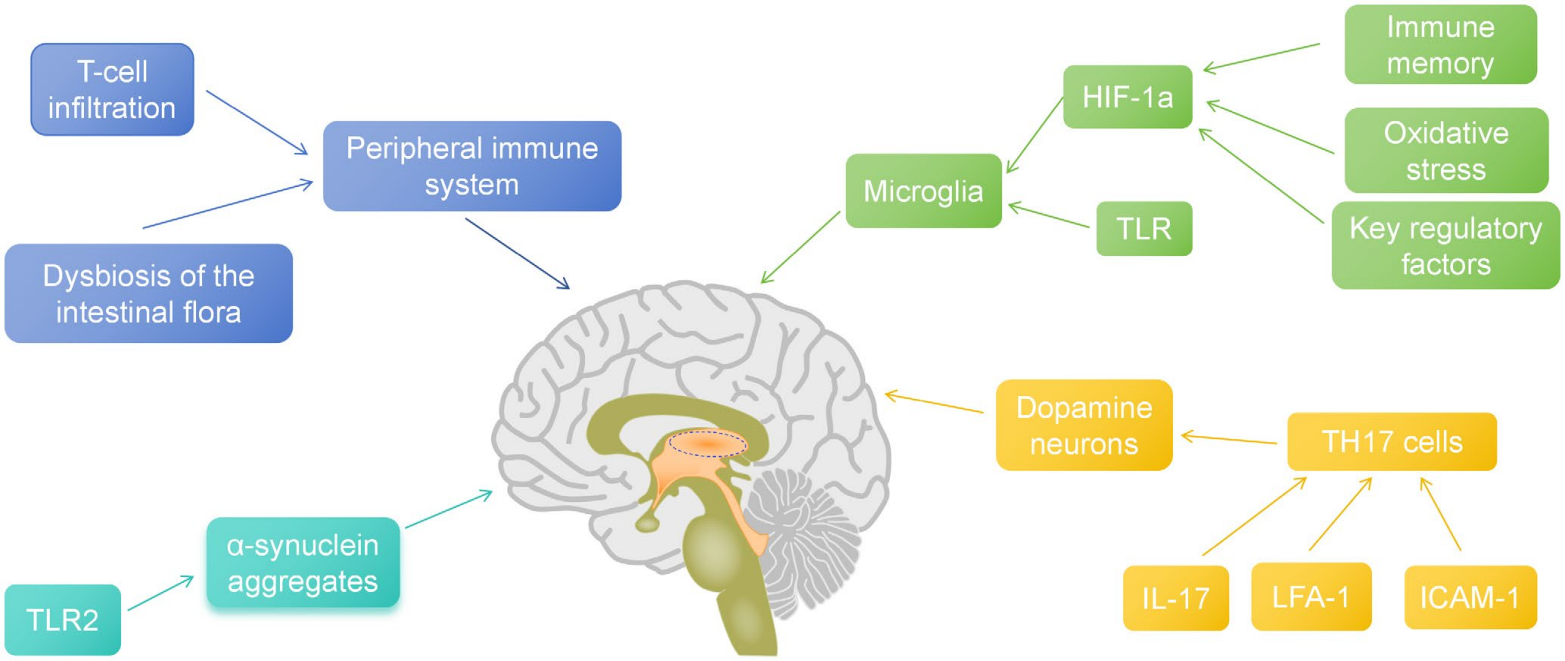
Neuroinflammation and α‐syn–driven microglial activation in PD. (1) α‐syn/microglia axis: α‐syn activates microglia via TLR2/4 and FcγR, triggering the NLRP3/caspase‐1 inflammasome pathway. (2) Inflammatory amplification: Pro‐inflammatory cytokines (IL‐1β, IL‐18) drive T‐cell involvement and neurotoxic (A1) astrocyte polarization. (3) Neurodegeneration: Persistent oxidative stress and mitochondrial dysfunction culminate in dopaminergic cell loss. (4) Therapeutic intervention: α‐syn immunotherapy clears aggregates, dampening the neuroinflammatory cascade and preserving neuronal integrity. FcγR, Fcγ receptor; IL‐1β interleukin‐1β; IL‐18 interleukin‐18; NLRP3, NOD‐like receptor protein 3.

PD‐related α‐syn fibrils and chronic inflammatory signals cooperatively induce microglia polarization toward a neurotoxic phenotype characterized by “high glutamate release and high iron retention,” which activates NMDA receptors in dopaminergic neurons and ultimately leads to excitotoxic death [61]. STING is a key α‐syn PFF‐induced nigrostriatal damage. α‐syn–PFF activates the cGAS/STING pathway by triggering double‐strand breaks in microglial DNA, driving type I interferon inflammatory responses, and ultimately leading to the death of dopaminergic neurons; STING‐deficient mice are completely resistant to α‐syn–PFF‐induced motor disorders, pathological aggregation and neuronal loss, and the level of STING protein in the substantia nigra of human PD patients is significantly positively correlated with the degree of α‐syn pathology [62] (see Table 1 and Figure 2).

**TABLE 1:** The role of immunity on pathogenesis of PD.

Components of the immune system	The role in PD	Relevant mechanisms	Data analysis	Discussion of limitations	References
Microglia	The main players in neuroinflammation are mediated by HIF‐1α and TLR pathways	HIF‐1α regulates immune memory and oxidative stress. TLR2 recognizes α‐syn aggregation and triggers inflammatory response	In the cerebrospinal fluid of PD patients, the concentrations of proinflammatory factors (TNF‐α, IL‐1β, IL‐6) secreted by pro‐inflammatory microglia were 1.8 times, 2.5 times, and 1.6 times higher than those in healthy individuals, respectively. In the in vitro culture system of microglia stimulated by α‐syn PFF, the release of IL‐1β reached its peak at 24 h after stimulation, which was 4.2 times that of the control group	Most existing PD models (such as MPTP and α‐syn PFF injection) are acutely induced and cannot fully simulate the chronic progression process of human PD. Microglia cultured in vitro (such as BV2 cells) lack the complex neural microenvironment in vivo (such as neuron–glia interaction and the blood–brain barrier), making it difficult to accurately reflect their true functional state in PD	[63, 64, 65, 66, 67, 68]
HIF‐1α	Regulate the innate immune response of microglia and participate in the pathological mechanism of PD	HIF‐1α regulates cellular metabolism and functions through the NF‐κB signaling pathway, and promotes the expression of inflammatory factors (such as IL‐1β)	A mouse model was established by intraperitoneal injection of different doses of LPS (1xLPS to induce immune training TR and 3xLPS to induce immune tolerance TL). It was found that the proportion of HIF‐1α^+^ microglia in the striatum of the TR group was 20%–30% higher than that of the TL group (immunofluorescence counting, *p* < 0.01); and the mRNA expression levels of pro‐inflammatory factors (IL‐1β, IL‐6, TNF‐α) in the TR group were 1.8 times, 1.6 times, and 1.7 times those of the TL group, respectively (RT‐qPCR, *p* < 0.05)	The expression of HIF‐1α is correlated with inflammatory factors and neuronal injury, but there is a lack of direct evidence to prove that HIF‐1α is the key factor regulating these processes	[69, 70, 71, 72, 73, 74, 75, 76, 77, 78, 79, 80]
Toll‐like receptors (TLRs)	Trigger neuroinflammatory responses and recognize α‐syn aggregation as damage‐associated molecular patterns (DAMPs)	TLR2 is upregulated in microglia and neurons, activating pro‐inflammatory signaling pathways and leading to neuroinflammation	In the brain tissues of deceased PD patients, the expression levels of TLR2 mRNA in the substantia nigra (SN), caudate nucleus and putamen regions were 1.8–2.3 times higher than those in healthy individuals (RT‐qPCR, *p* < 0.01), and the expression of TLR2 in neurons was positively correlated with the aggregation amount of α‐syn (Pearson correlation coefficient *r* = 0.68, *p* < 0.01)	Most quantitative data on TLRs come from animal models, while the sample size of studies on PD patients (such as cerebrospinal fluid and postmortem brain tissue) is relatively small	[81, 82, 83, 84, 85, 86, 87, 88]
TH17 cells	Induction of dopaminergic neuron death through IL‐17 and LFA‐1/ICAM‐1 pathways	TH17 cells secrete IL‐17, which directly interacts with ICAM‐1 on neurons, leading to neuronal damage	In the PD model constructed with human‐induced pluripotent stem cells (iPSCs), the proportion of patient‐derived peripheral blood Th17 cells was 2.3 times higher than that of the healthy control group; the number of Th17 cells in the cerebrospinal fluid was 1.8 times that of healthy people	The difference in pathology between animal models and humans: Th17 cell infiltration shows a rapidly increasing trend in animal models, while in human PD, Th17 cell infiltration is a progressive process	[89, 90, 91, 92]

*Note:* 1. This study focuses on the role of immune system components in the pathogenesis of PD, centering on four core components: microglia, HIF‐1α, TLRs, and TH17 cells. 2. It clarifies the functions of each component, such as microglia being the main mediators of neuroinflammation and TH17 cells inducing the death of dopaminergic neurons; and elaborates on key mechanisms, including HIF‐1α regulating the expression of inflammatory factors through the NF‐κB pathway and TLR2 recognizing aggregated α‐syn to trigger inflammation. 3. At the same time, it objectively points out the limitations of the research, such as most PD models being unable to simulate the chronic course of the disease in humans and the small sample size of TLRs‐related patient samples.

### 3.2 Passive Immunotherapy With α‐Syn–Directed Antibodies

Blocking “prion‐like” transmission: Pathological α‐syn aggregates, especially oligomers, can spread between neurons, inducing normal proteins to misfold in a domino‐like manner. Passive immunotherapy prevents the uptake of extracellular α‐syn aggregates by neighboring neurons through antibody binding, thereby interrupting the spread of the pathological process [93]. Antibody binding to pathological proteins can also activate microglia through their Fc segments, promoting phagocytosis and clearance of protein aggregates. The toxicity of α‐syn oligomers to organelles such as mitochondria and the endoplasmic reticulum is a key contributor to neuronal death [94].

Alternative therapies, including gene silencing techniques, use antisense oligonucleotides or small‐interfering RNA (siRNA) to reduce α‐syn protein expression at the source. The nanomedicine called REXO‐C/ANP/S has successfully cleared α‐syn and improved motor function in animal models. Clearance can also be achieved by using small molecule drugs (such as curcumin) to directly degrade the formed aggregates, often in combination with gene therapy to create a synergistic effect [95]. Clinical trials usually intervene only after patients have developed obvious motor symptoms, by which time the neurodegeneration has become so severe that it is difficult to reverse. Therefore, the success rate of passive immunotherapy clinical trials is extremely low.

A copious amount of evidence indicates that immune system disorder exerts an influence on PD. These findings involve multiple areas: clinical and genetic links between autoimmune diseases and PD, abnormal cell and fluid immune functions found in PD patients, activated inflammatory cells confirmed by imaging studies in PD, and immune dysregulation associated with PD observed in experimental models [96, 97]. Despite the fact that the relationship between the immune system and PD is not yet fully understood, and the specific roles of innate immunity and adaptive immunity in neurodegenerative diseases have not yet been definitively established, research in this area is gradually deepening, current research provides a theoretical basis for targeted immune system treatments. These potential therapies are expected to be validated in patients with PD. Currently, several clinical trials are underway to evaluate the effectiveness of these treatment options [98, 99].

**FIGURE 3:**
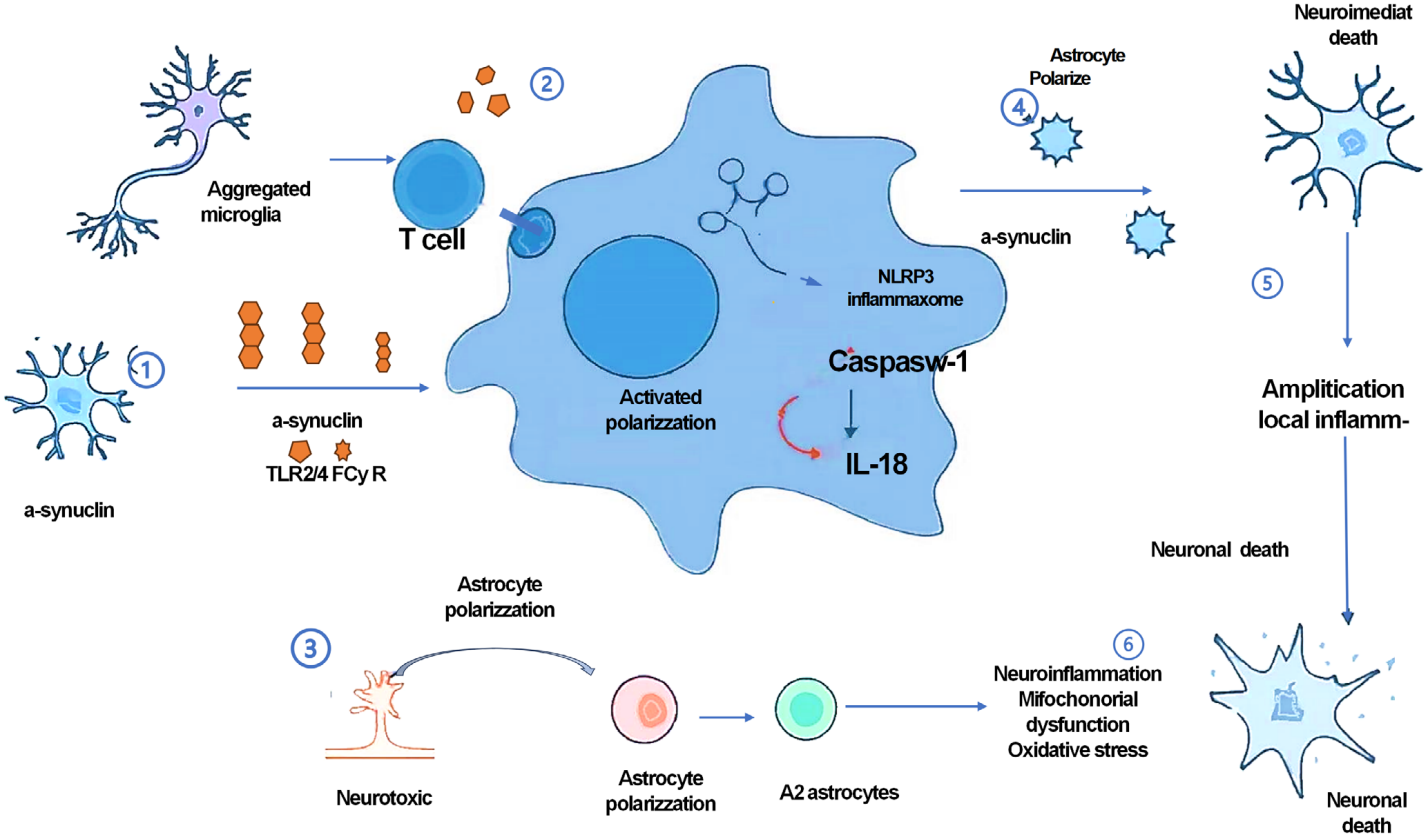
Microglia‐mediated neuroinflammation via HIF‐1α and TLR pathways. Key regulator of immune memory, oxidative stress, and interaction with PD‐related genes. TH17 cells induce dopaminergic neuron death through IL‐17 and LFA‐1/ICAM‐1 pathways. Peripheral immunity impacts PD progression: Notable features include T‐cell infiltration and gut dysbiosis. TLR2 detects α‐syn aggregates, triggering inflammatory cascades in PD brains. HIF‐1α, hypoxia‐inducible factor‐1a; IL‐17, interleukin 17; LFA‐1, function‐associated antigen; TLR, Toll‐like receptor; TH17, T helper 17; TLR2, Toll‐like receptor 2; 1 ICAM‐1, intercellular adhesion molecule 1.

There is a broad consensus that the immune system plays an indispensable and crucial role in the development, homeostasis maintenance and functional realization of the central nervous system (CNS), functioning as an invisible guardian that silently regulates the delicate balance and efficient operation of the nervous system, with the innate immune cells and the related signaling molecules being major participants [100, 101] (see Table 2 and Figure 3).

**TABLE 2:** Summary table of core information on the association between PD and immunity.

The dimension of PD's association with immunity	Core conclusions	Key evidence	References
Autoimmunity and PD	Autoimmune abnormalities (T‐cell activation, autoantibodies, genetic associations) are involved in the pathogenesis of PD	1. T cells of PD patients are activated by α‐syn, and SNCA aggregation activates astrocytes	[88, 102, 103, 104, 105, 106, 107]
		2. Anti‐α‐syn autoantibodies were detected in the patient's serum/cerebrospinal fluid, and IgG damaged dopaminergic neurons through Fcγ receptors	
		3. HLA‐DRB104:01/11:01 is a genetic risk marker for PD, and HLA‐DRB104 can activate protective CD4^+^ T cells	
Central immunity and PD	Central α‐syn aggregation triggers neuroimmune responses, and immunotherapy has the potential to modify the disease	1. Lewy bodies (α‐syn aggregation) activate microglia and T cells, and the imbalance of Teffs and Tregs leads to neuronal degeneration	[108, 109, 110]
		2. Familial PD is associated with point mutations/gene duplications of α‐syn	
		3. Targeted α‐syn immunotherapy and MSCs therapies such as OM‐MSCs show potential, and the immune strategies for MS may be borrowed	
Peripheral immunity and PD	Peripheral immune activation is involved in the progression of PD, is associated with clinical features and shows gender differences. “Body‐predominant” PD is related to peripheral factors	PD may progress from the periphery to the central nervous system, and there is infiltration of CD4^+^/CD8^+^ T cells in the patient's brain	[111, 112, 113, 114, 115, 116]
		The proportion of CD4^+^ T cells in female patients was negatively correlated with the Hoehn–Yahr stage, while the IgG level was positively correlated with the disease duration/UPDRS III score	
		“Body‐first” PD begins in the gut, accompanied by dysbiosis of the intestinal flora	

*Note:* 1. From the perspectives of autoimmunity, central immunity, and peripheral immunity, the core information on the association between PD and immunity is sorted out. 2. The autoimmunity dimension points out that autoimmunity abnormalities (T‐cell activation, autoantibodies, etc.) are involved in the onset of PD, listing key evidence such as the activation of T cells in PD patients by α‐syn; the central immunity dimension indicates that the aggregation of central α‐syn triggers neuroimmune responses, and immunotherapy has the potential to modify the disease, mentioning the activation of immune cells by Lewy bodies and other contents; the peripheral immunity dimension proposes that peripheral immune activation participates in the progression of PD and there are gender differences, “body‐predominant” PD is related to peripheral factors, and provides evidence that PD may progress from the periphery to the center, clearly demonstrating the multi‐faceted nature of the association between PD and immunity.

### 3.3 Microglia

Previous studies have indicated that microglia, as the main innate immune cells in the brain, possess the ability to store innate immune memory (IIM) [63, 64, 65]. Microglia, as the resident macrophages in the CNS, play a pivotal role in neuroinflammation, acting as invisible regulators and profoundly influencing the pathological progression of PD [66, 67]. In the early stage of PD, microglia are mainly of the anti‐inflammatory which can secrete IL‐10, TGF‐β, and other factors to protect neurons. As the disease progresses, they are stimulated by α‐syn and neurotoxins (such as MPTP, 6‐OHDA) and polarize to the pro‐inflammatory, releasing TNF‐α, IL‐1β, and other factors to trigger neuroinflammation and accelerate dopaminergic neuron death. Meanwhile, IL‐1α, IL‐1β, and other factors secreted by pro‐inflammatory microglia can activate neurotoxic A1‐type astrocytes, jointly exacerbating neural damage, whereas anti‐inflammatory microglia can induce protective A2‐type astrocytes, forming a neuroprotective mechanism [68].

### 3.4 Hypoxia‐Inducible Factor‐1a (HIF‐1a)

Research has found that the absence of HIF‐1a in microglia can alleviate motor dysfunction and reduce neuronal damage in mice with PD to a certain extent. The experimental results suggest that HIF‐1a within microglia may become a potential regulatory target of innate immune responses and play a significant role in the pathological mechanism of PD [69].

With the deepening of research, more and more evidence indicates that HIF‐1a plays a key role in the pathological processes of various diseases. Inflammatory responses, infection processes, and the occurrence of cancer can also promote the stable expression of HIF‐1a [69, 70, 71, 72]. Studies have shown that HIF‐1a plays a significant role in the activation and polarization of macrophages. It regulates cellular metabolism and function by interacting with the nuclear factor‐κB (NF‐κB) signaling pathway, thereby promoting the expression of various inflammatory cytokines, including interleukin‐1β (IL‐1β) [73, 74]. In macrophages, the absence of HIF‐1a has been confirmed to weaken the inflammatory response and may affect the function of IIM [75]. The increased expression of HIF‐1a is closely related to the disease progression of patients with PD [76]. In the pathological mechanism of PD, the transcription factor HIF‐1α plays a crucial role in the disease progression by regulating the expression patterns of its downstream genes [76]. HIF‐1a has been found to be involved in the oxidative stress associated with PD pathogenesis and has been shown to interact with multiple PD causative genes, such as DJ‐1, PINK1, and ATP13A2 [76]. As a transcription factor, it could regulate the expression of PD‐predicted pathogenic genes such as DJ‐1, PINK1, and ATP13A [77, 78, 79]. However, during the pathological process, the specific mechanism by which HIF‐1a regulates inflammasomes in microglia remains unclear [69]. Several studies have reported that HIF‐1α played a crucial role in immune‐inflammatory response [70, 80].

### 3.5 Toll‐Like Receptors (TLRs)

TLRs are important receptors in the innate immune system and are widely distributed in various cells and tissues of the human body [81]. In the pathological mechanisms of various CNS diseases, TLRs may exhibit dual effects, having both potential protective roles and the possibility of causing adverse effects [82]. The latest research indicates that TLRs may play a crucial immune‐mediated role in PD, as these receptors can trigger neuroinflammatory responses [83]. When TLRs bind to their corresponding ligands, they activate downstream signaling pathways, ultimately triggering a variety of cellular responses. For instance, TLRs may function by recognizing α‐syn aggregates as DAMPs. This recognition can activate pro‐inflammatory signaling pathways, thereby leading to the occurrence of neuroinflammation [83, 84].

Numerous studies have consistently demonstrated that postmortem analysis of brains from PD patients reveals upregulated expression of TLR2 [85, 86]. TLR2 is mainly expressed on microglia, and the neuronal expression of this receptor may have the potential to serve as a specific biomarker for PD [84]. TLR2 expression is positively correlated with amoeboid microglia in the hippocampus and substantia nigra of PD patients, indicating its role in microglia‐mediated neuroinflammation. Meanwhile, new evidence shows that TLR2 in astrocytes also participates in disease‐induced neuroinflammation by enhancing the inflammatory response [87, 88].

### 3.6 TH17

According to existing research, TH17 cells exacerbate dopaminergic neuronal loss in PD [89], TH17 cells can cause the death of dopaminergic neurons through two main mechanisms. First, lymphocyte function‐associated antigen 1 (LFA‐1) on the surface of TH17 cells can directly interact with intercellular adhesion molecule 1 (ICAM‐1) on neurons. Second, TH17 cells can secrete interleukin 17 (IL‐17), thereby affecting neuronal survival. Both pathways may contribute to dopaminergic neuron damage [90, 91]. Therefore, counteracting TH17 cell development or blocking the interactions between LFA‐1 and ICAM‐1, or IL‐17 and its receptor, could serve as potential therapeutic strategies in PD [92] (see Figure 2).

## 4 Immunotherapeutic Approaches to PD

### 4.1 Autoimmunity and PD

Several studies have shown that PD is associated with dysregulated innate and adaptive immune responses. Recent studies have shown that T lymphocytes in patients with PD can be activated byα‐syn, suggesting that autoimmune inflammation may play a role in the pathological process of the disease. This result indicates that autoimmune responses may occupy an important position in the pathogenesis of PD [102]. Autoantibodies targeting α‐syn have been detected in both the serum and cerebrospinal fluid (CSF) of patients with PD [88, 103]. At the core of the autoimmune response taking place in PD is SNCA. Studies have shown that when this protein forms a cluster and avoids degradation by the proteasome and autophagosomes, it activates a specific type of cell—astrocytes (macrophages). These cells make up 10%–15% of the brain. This condition critically alters the neurotransmitter pathways. The prevalent immunopathological features within the substantia nigra in PD consist of activated microglia, the expression of MHC‐I and MHC‐II alleles, modified antigen presentation, and an enhanced pro‐inflammatory cytokine profile, all contributing to dysregulated activity of the innate and adaptive immune system and to typical self‐tissue damage [104]. Astrocytes, as the most numerous type of cells in the central nervous system, also play a crucial role in the inflammatory response of the brain. Research has found that immunoglobulin (Ig)G can activate these cells through the Fcγ receptor (FcRγ) pathway in PD patients, leading to damage to the dopaminergic neurons in the substantia nigra region. Multiple in vitro and in vivo experiments have further confirmed that synthetic antibodies may have adverse effects on the dopamine system. These engineered antibodies can specifically bind to native dopamine receptors and competitively inhibit the binding of their natural ligands, consequently disrupting the normal function of the dopamine system [105].

Studies have found that HLA‐DRB1 alleles, especially DRB104:01 and DRB111:01, can serve as core markers for PD genetic risk prediction, and combining with other genes (such as LRRK2 and SNCA) can improve the accuracy of risk assessment [106].

The H13/H33 site‐regulated peptide binding ability of HLA‐DRB1*04 specifically recognizes the acetylated tau PHF6 sequence and activates protective CD4^+^ T‐cell responses, thereby reducing the risk of PD and slowing pathological progression [107].

### 4.2 Central Immunity and PD

Lewy bodies that appear in the cytoplasm of the brain are one of the key pathological features of PD. These Lewy bodies are mainly composed of aggregates of α‐syn. In the CNS, the aggregation of α‐syn can activate microglia cells, thereby triggering neuroinflammatory responses. Additionally, these aggregates can also activate the antigen‐presenting pathway to reactivate T cells, ultimately triggering a neuroimmune response against dopaminergic neurons. The abnormal activation of microglia and the infiltration of T cells constitute the core effector of the immune response. The imbalance of Teffs–Tregs further amplifies the immune‐mediated damage and eventually leads to the degeneration of dopaminergic neurons. Based on this mechanism, immunotherapy targeting α‐syn and multi‐target immunomodulatory therapy of mesenchymal stem cells (MSCs) provide a key strategy for disease‐modifying treatment in PD. Among these, olfactory mucosa MSCs (OM‐MSCs) have shown considerable clinical potential due to their convenient source, low immunogenicity, and age stability [108]. Lewy neurites stand for filamentous neuritic inclusions within degenerating dopaminergic neurons. In familial PD, researchers have identified multiple point mutations in the α‐syn protein, including A30P, E46K, H50Q, G51D, A53E, and A53T. Additionally, rare genetic duplications or trisomies have been found, which result in two additional autosomal dominant or recessive forms of PD. Studies on the neuroinflammatory pathology of human PD have shown a wide range of expression patterns. This might partly be because the clinical diagnosis of PD still has uncertainties [109]. In recent years, an increasing amount of research evidence has indicated that inflammation plays a crucial role in the occurrence and development of neurodegenerative diseases in the CNS. This realization has prompted researchers to explore more immune‐regulatory strategies to combat diseases related to neuroinflammation. A considerable number of these approaches that have been inspected in multiple sclerosis (MS) might also prove efficacious in other neurodegenerative disorders, as evidenced by Gilenya (FTY720, S1P‐R agonist) for experimental PD [110]. In the future, it will be necessary to further explore the dynamic relationship between central immunity and the pathological progression of PD, and to optimize immunotherapy regimens to achieve effective prevention and control of PD.

### 4.3 Peripheral Immunity and PD

The potential contribution of peripheral immunity in neuroinflammation and neurodegeneration has raised increasing interest only during the last decade. In contrast with the profusion of studies available on neuroinflammatory processes that take place in the CNS during the advancement of PD, the specific role of the peripheral immune system during PD is still weakly understood. Indeed, studying the role of peripheral immunity in PD is difficult, a further complication arising from the complex interrelation among PD, aging and immunity [111]. In consonance with the theory of Braak's staging in PD, the pathogenesis is manifested to progress from the peripheral system to the CNS, and peripheral immune activation has been hypothesized to have a role in the etiology of PD. In the brains of deceased PD patients and animal models, there have been observations of T‐cell infiltration. The lymphocytes that enter the brain form a heterogeneous group, which includes both CD4+ and CD8+ cell types. In this review regarding the evidence of peripheral immune activation in PD. The augmented immune activation in the peripheral system is manifested in PD, and dynamic alterations in the percentage of CD4+ T cells and the level of IgG imply an active role of peripheral immunity in the disease progression, particularly in female PD patients. The percentage of CD4^+^ T cells in PD patients was negatively correlated with H&Y stage (r = −0.121, *p* = 0.024), and this association was only observed in female patients (r = −0.201, *p* = 0.011 for female patients, but not in male patients). Serum IgG level was positively correlated with the duration of PD (*r* = 0.036, *p* = 0.011) and UPDRS III score (*r* = 0.117, *p* = 0.035), that is, the longer the disease duration and the more severe the motor symptoms, the higher the IgG level. Again, this association was significant only in female patients (female disease duration *r* = 0.173, *p* = 0.029) [112]. Recent clinical and imaging studies support the occurrence of “body‐first” PD, presumed to start in peripheral organs such as the gut, and reveal unique characteristics, including gut dysbiosis, for this type of PD, which is thought to initially appear in peripheral sites rather than in the CNS [113].

The traditional view holds that the disruption of the blood–brain barrier (BBB) is a consequence of the disease's advanced stage. However, an increasing body of evidence suggests that immune cells can infiltrate the CNS during the prodromal or early stages, not as a passive “invasion after barrier breakdown,” but as an active, multi‐step process. Studies have indicated that both neuroimaging and autopsy results show that patients with PD may have BBB dysfunction or increased permeability in the early stages of the disease, providing conditions for the infiltration of peripheral immune cells [114]. Research indicates that only a portion of patients with PD have peripheral T cells that respond to α‐syn (referred to as PD responders, PD_R). One study found that in these patients, a specific CD4+ T‐cell subset—Granulysin+ cytotoxic effector memory T cells—is significantly reduced. This suggests that future immunotherapy may need to be preceded by immune typing, with medication administered only to PD_R patients to avoid unnecessary risks to nonresponders (PD_NR) [115]. Precisely targeting the pathological forms of α‐syn and reducing cross‐reaction with normal proteins can be achieved through careful selection of T‐cell epitopes. The immunodominant epitopes of α‐syn (such as amino acids 15–23 and 35–55) are exposed in the pathological aggregated state but hidden in the physiological monomer. By designing peptide vaccines (such as PD01A) targeting these “pathology‐specific epitopes,” CD4^+^Treg cells that only recognize pathological α‐syn can be activated, rather than pro‐inflammatory Th1/Th17 cells [116] (see Table 2).

### 4.4 Familial PD and Sporadic PD

Familial or sporadic PD is often associated with specific gene mutations. Neuronal damage in familial PD may be more dominated by metabolic dysfunction within cells, which limits the efficacy of antibody therapies that primarily act extracellularly [117]. For more common sporadic PD, although genetic factors are not prominent, adaptive immunity (especially T cells) appears to play a more central role. A notable feature of sporadic PD is the involvement of the “gut‐brain axis.” Interventions targeting the gut microbiome, such as probiotics or fecal microbiota transplantation, may be particularly beneficial for patients with sporadic PD [118].

## 5 Immunotherapy

Immunotherapy can be divided into two categories based on its mechanism of action: active immunotherapy, which relies on the stimulation of specific antigens to work, and passive immunotherapy, which is primarily achieved through the infusion of exogenous antibodies. Active immunotherapy evokes the host immune response, leading to the generation of antibodies and activation of T cells [119].

### 5.1 Active Immunization

Active immunotherapy approaches in the field of Lewy body disease are becoming a research hotspot and are regarded as a promising treatment option [120]. An effective active immunization strategy in PD must ensure sufficient antibody transfer across the BBB [121]. Active immunization elicits an immune response and the formation of antigen‐specific antibodies. Immunogen constructs can therefore be engineered against epitopes of pathologic, aggregation‐prone variants of Aβ, Tau, or α‐syn to avoid interfering with normal physiology [122].

Active immunotherapy for Alzheimer's disease (AD) targets two key proteins: beta‐amyloid (Aβ) and microtubule‐associated protein Tau. In contrast, active immunotherapy for PD focuses on a single core target: α‐syn [123]. Both aducanumab for AD and cinpanemab for PD can reduce the load of target proteins, but they have not led to significant improvements in cognitive or motor functions. The low penetration rate of the BBB and the relatively large molecular weight of most mAbs, such as the CSF/serum ratio of cinpanemab, result in insufficient drug concentration in the brain, making it impossible to effectively clear the pathological proteins in the CNS [124] (see Table 3).

**TABLE 3:** Clinical trials of immunotherapy strategies for PD.

Classification of immunotherapy	Types of immunotherapy	Target	Clinical trial number	The test subjects	Clinical trial stage	Trial endpoint	Result	References
Active immunotherapy	PD01A (ACI‐7104)	Polymeric α‐syn oligomers	NCT02267434	24 patients with early stage PD	Phase I completed; randomized, multicenter, parallel, placebo‐controlled, patient‐blinded study to monitor the tolerability and safety of the drug formulated with an adjuvant for patients suffering from early PD. Both low and high doses were systemically and locally well tolerated. Renamed as ACI‐7104.056 (AC Immune), a phase II study (NCT06015841) has been initiated	The motor function was evaluated using the third part of the MDS‐UPDRS, suggesting that the drug had no significant short‐term effect on motor function	Trigger a significant humoral immune response, effectively bind to the target, and have good safety profile	[125, 126]
Active immunotherapy	PD03A	C‐terminal α‐syn epitope	EudraCT 2014‐000568‐16	36 patients with early PD	Completed, Phase II clinical trial	Motor function: The MDS‐UPDRS I‐III sections were scored (ON state), indicating that the drug did not significantly affect the progression of motor function. Nonmotor symptoms and quality of life: There were no significant differences between groups in the NonMotor Symptoms Scale (NMSS), PD Questionnaire‐39 (PDQ‐39), and cognitive tests (such as MoCA and Wisconsin Card Sorting Test).	The safety profile is good and the antibody response is significant. This validates the potential application value of the SAIT method in the treatment of PD	[127]
Active immunotherapy	UB‐312	Polymeric and fibrillar α‐syn oligomers and fibrils	NCT04075318	50 healthy volunteers	Completed	No MDS‐UPDRS score	High safety profile, good tolerance, time‐dependent and dose‐dependent anti‐α‐syn antibody production	[126, 128, 129]
Passive immunotherapy	ABBV‐0805	Aggregated α‐syn	NCT04127695	Unknown	Phase I trial stage	Unknown	Test status: Withdrawn	[130, 131]
Passive immunotherapy	BIIB054 (cinpanemab)	Aggregated α‐syn	NCT03318523 (SPARK)	Unknown	The Phase II study (SPARK, NCT03318523) failed and was suspended. Further development was halted.	Primary endpoint was to measure the MDS‐UPDRS score, secondary outcome was to evaluate the serum concentration and SBR in the putamen, as measured by SPECT‐DaT	It has a high affinity for fibrillar α‐syn and is safe, but the Phase II trial failed to reach the primary endpoint	[132, 133, 134, 135]
Passive immunotherapy	Prasinezumab (PRX002)	The C‐terminal of α‐syn protein	NCT02095171	40 healthy volunteers	Phase I clinical trial has been completed	Not involving MDS‐UPDRS scores	The safety profile is good. It can reduce the level of free α‐syn in serum, but it shows no significant improvement in motor symptoms	[136]
			NCT02157714	60 patients with mild to moderate PD	In a phase Ib trial, Completed	The MDS‐UPDRS did not improve, but the safety of the drug was confirmed.	The research period is relatively short	[137]
			NCT03100149 (PASADENA)	316 patients with PD	Phase II ongoing	There was no significant difference in the MDS‐UPDRS scores between Part 1 (0–52 weeks) and Part 2 (56–104 weeks) of the trial	Preliminary data show that there were no significant differences between the active drug group and the placebo group in any of the prespecified endpoint indicators. One death (suicide) occurred 26 days after the first administration (1500 mg)	[138]
			NCT04777331 (PADOVA)	410 patients with early PD	Phase IIb, Completed	The 4500 mg prasinezumab group significantly delayed the deterioration of the total MDS‐UPDRS score compared to the placebo group, while no significant difference was observed in the 1500 mg group, suggesting that a high dose has the potential for disease modification	Only 8 cases (2%) of patients developed anti‐prasinezumab antibodies, with no antibody‐related adverse events, indicating a low immunogenicity risk	[119, 139]
Passive immunotherapy	MEDI1341	α‐syn	NCT04449484	Twenty‐five patients with mild to moderate PD	Completed	Not involving MDS‐UPDRS scores	It can enter the brain through intravenous injection and effectively reduce the level of α‐syn protein	[126]
			NCT03272165	49 healthy volunteers	Phase I completed, The second phase study (NCT05526391) for patients with multiple system atrophy (MSA) has been initiated	Not involving MDS‐UPDRS scores		[140]

*Note:* 1. The core information of clinical trials on immunotherapy for PD was systematically summarized and classified into two major categories: active immunotherapy and passive immunotherapy. 2. Active immunotherapy involves PD01A (targeting α‐syn oligomers), PD03A (targeting the C‐terminal epitope of α‐syn), UB‐312 (targeting aggregated and fibrillar α‐syn), etc. The results show that this type of therapy can generally trigger significant antibody responses and is safe, but some have no short‐term improvement effect on motor function. 3. Passive immunotherapy includes ABBV‐0805 (targeting aggregated α‐syn), BIIB054 (targeting aggregated α‐syn, suspended after the phase II trial failed to meet the primary endpoint), prasinezumab (targeting the C‐terminal of α‐syn, high dose or delays symptom deterioration), MEDI1341 (can enter the brain and reduce α‐syn levels), etc. This demonstrates that passive immunotherapy has some failed trials, but some therapies have the potential to modify the disease.

#### 5.1.1 PD01A

PD01A is a specific active immunotherapy with a short peptide formulation targeted against oligomeric α‐syn. In the initial phase of the study, we examined the safety and tolerability of PD01A immunotherapy in patients with PD. The specific active immunotherapy triggered a significant humoral immune response and successfully achieved an effective binding to the target. In order to more comprehensively evaluate the safety and efficacy of PD01A in the treatment of PD, a second‐stage study is necessary [125]. In 2023, AC Immune SA launched a new multi‐center Phase II clinical trial to evaluate the safety, tolerability, immune response, and pharmacokinetic profile of PD01A in early PD patients (NCT06015841). This trial will be completed in 2028 [126].

#### 5.1.2 PD03A

This synthetic peptide antigen (PD03) consists of 10 amino acids and mimics an epitope in the C‐terminal region of α‐syn. In this study, PD03A demonstrated excellent safety and significant antibody responses, further validating the potential application value of the SAIT method in Phase II clinical trials for PD [127].

#### 5.1.3 UB‐312

UB‐312 is an α‐syn peptide conjugated to a T helper peptide and is expected to induce antibodies specifically against oligomeric and fibrillar α‐syn, making UB‐312 a potential immunotherapeutic for synucleopathies. In this first‐in‐human study, UB‐312 was generally safe, well‐tolerated, and generated robust time‐ and dose‐dependent anti‐α‐syn [128]. Currently, UB‐312 is conducting a Phase 1 clinical trial to evaluate the safety and tolerability of the vaccine in healthy individuals and mild PD patients (NCT04075318) [129]. Additionally, another Phase 1b clinical trial for UB‐312 (NCT05634876) is also ongoing and currently recruiting participants. The main objective of this study is to evaluate the safety, tolerability, and immune response induced by the drug in PD patients, and the study is expected to be completed by 2025 [126].

### 5.2 Passive Immunity

Passive immunization induces clearance of toxic α‐syn aggregates from neuronal populations by administering anti‐α‐syn–directed antibodies to the CNS [141]. Currently, the best‐case scenario for passive immunization is a modest, short‐term reduction in clinical decline that may offer several months or years of better‐quality life [123].

#### 5.2.1 ABBV‐0805

ABBV‐0805, a monoclonal antibody, shows high selectivity toward human aggregated α‐syn and has an extremely low affinity for monomers. ABBV‐0805 binds to broad‐spectrum soluble aggregated α‐syn. ABBV‐0805 has been progressed into clinical development as a potential disease‐modifying treatment for PD patients [130]. Early clinical data suggest that ABBV‐0805 exhibits high selectivity for human α‐syn aggregates, while its binding ability to monomers is relatively weak. In mouse experimental models, treatment with mouse homologs resulted in improved pathological changes and spread of α‐syn, as well as an extended lifespan in mice. The in vitro binding of ABBV‐0805 to pathological α‐syn was validated in the postmortem brains of patients with PD [131].

#### 5.2.2 BIIB054 (Cinpanemab)

BIIB054 is a human‐derived α‐syn antibody that specifically binds to aggregated forms of α‐syn. The affinity of this antibody for fibrillar α‐syn is much greater than its affinity for monomeric recombinant α‐syn, with a difference in affinity of over 800‐fold [132]. Additionally, BIIB054 shows selectivity only for the aggregated form of α‐syn, suggesting that this antibody may result from the immune response of the body to misfolded α‐syn [133]. BIIB054 has good safety, tolerability, and pharmacokinetics, which is beneficial for PD patients. In PD patients, BIIB054 has demonstrated a high binding ability to α‐syn, approaching maximum complex formation levels. The drug has been tested in two separate trials at different dosages, and preliminary trial results suggest that it may show potential therapeutic effects in the first stage of clinical trials. It is worth noting that specific data from the study have not yet been released, and the drug has progressed to the second stage of clinical trials [134]. A phase II study (SPARK, NCT 03318523) failed to meet its primary and secondary endpoints, prompting the suspension of further cinpanemab development. The reasons for its failure include, from the “time dimension,” the intervention being too late to reverse existing neuronal damage, as it occurred after the initiation stage of α‐syn pathology. From the “mechanism dimension,” it targeted extracellular aggregated α‐syn, failing to address early intracellular pathology, and a single target was insufficient to counteract the multi‐pathway damage of the disease [135]. This failure transcends the limitations of a standalone drug, fundamentally challenging the validity of the ‘extracellular neutralization’ paradigm. While BIIB054 was engineered to intercept the trans‐synaptic spreading of proteopathic seeds, its inability to access intracellular pathogenic compartments rendered it ineffective against the entrenched pathology and concomitant neuronal attrition. These outcomes suggest that the future of immunotherapy lies in a strategic shift from monolithic targeting to multifaceted synergistic interventions, coupled with a concerted effort to overcome the diagnostic bottleneck of early preclinical detection.

#### 5.2.3 Prasinezumab (PRX002)

Prasinezumab is a humanized monoclonal antibody [142]. Prasinezumab was capable of slowing down motor deficits in patients with a more rapid disease progression, most probably because the beneficial effect could be measured in this situation [143]. In a phase I clinical trial (NCT02095171) conducted on 40 healthy volunteers, the study results showed that the drug had good safety and was able to effectively lower free α‐syn levels in the serum in a dose‐dependent manner [136]. In a phase Ib trial involving 60 patients with mild to moderate PD, the safety profile was confirmed, although no improvement was observed on the Movement Disorders Society Unified PD Rating Scale (MDS‐MDS‐RS) (NCT 02157714). This is due to the relatively short duration of the study [137]. Prasinezumab recognizes the C‐terminus of α‐syn and binds well to both aggregates and monomeric proteins, whereas cinpanemab recognizes the N‐terminus and binds with lower affinity to monomeric α‐syn. In a random phase II clinical trial (NCT03100149), the prasinizam treatment group did not show significant differences in the 1‐year progression of MDS‐UPDRS parts I, II, and III compared to the placebo group. The most frequently reported adverse event was infusion reactions [138]. Prasinezumab was capable of slowing down motor deficits in patients with a more rapid disease progression, most probably because the beneficial effect could be measured in this situation. A second phase IIb trial (PADOVA) is ongoing with intravenous prasinezumab (NCT 04777331) in patients with early PD [119, 139].

Unlike cinpanemab, prasinezumab is characterized by its C‐terminal engagement and potent binding to α‐syn monomers. Nevertheless, the suboptimal outcomes of the PASADENA study invite a critical re‐examination of this approach. It is hypothesized that the antibody's high monomeric affinity causes it to be sequestered by the peripheral physiological pool, thereby failing to intercept pathogenic species in the CNS. Furthermore, the perceived clinical signals in the fast‐progressing subgroup should be interpreted with caution, as post hoc stratifications are prone to capturing statistical artifacts rather than true biological effects. In this context, the ongoing PADOVA trial is pivotal, not only for its clinical endpoints but as a definitive evaluation of whether the C‐terminal epitope provides a superior mechanistic leverage compared to N‐terminal directed therapies.

#### 5.2.4 MEDI1341

The study found that MEDI1341 can enter the brain through intravenous injection and effectively reduce the level of α‐syn [144]. At present, MEDI1341 has only been confirmed to chelate extracellular α‐syn, while its effects on intracellular α‐syn remain unclear. Consequently, it is not yet possible to determine whether it influences the aggregation or clearance of intracellular α‐syn. Although inhibition of α‐syn propagation has been observed in animal models, the extent to which antibody‐mediated extracellular α‐syn clearance affects disease progression, as well as onset and duration of effect in humans, remains undefined. Additionally, animal studies are required to supplement PK/PD dynamic data and assess long‐term efficacy [143]. In 2022, AstraZeneca completed a multi‐center, randomized, double‐blind, placebo‐controlled study in PD patients of both sexes (NCT04449484) [126]. A Phase I trial (NCT03272165) began to administer a single escalating dose of MEDI1341 to 49 healthy volunteers. Each subject received a 60‐min intravenous antibody infusion or a placebo, followed by a three‐month observation period. The trial aimed to test up to six different doses, with the specific doses depending on the safety data [140]. MEDI1341 is the first anti‐α‐syn monoclonal antibody to achieve a reduction of over 50% in CSF‐free α‐syn, validating the pharmacodynamic potential of α‐syn–targeted therapy [145].

Although active immunization strategies (e.g., UB‐312, PD01A) can reduce CSF α‐syn aggregates/oligomers, and passive immunization (e.g., prasinezumab) can slow motor progression, most trials have failed to achieve the primary endpoint of “significant improvement on clinical scales.” This suggests that the association between α‐syn clearance and symptom relief requires further clarification. Antibodies induced by active immunization have difficulty penetrating the cell membrane and cannot effectively target intracellular α‐syn. Similarly, the low penetration rate of passive immunization antibodies through the BBB limits their efficacy within the central nervous system [140] (see Table 3).

### 5.3 The Challenge of BBB Penetration and Delivery Strategies

The suboptimal clinical outcomes of a‐syn–targeted antibodies, such as cinpanemab, are inextricably linked to the formidable restrictive properties of the BBB [123]. The intrinsic brain penetrance of monoclonal antibodies (mAbs) remains a primary pharmacokinetic bottleneck, often resulting in CNS drug concentrations that fall below the threshold required for effective aggregate clearance [146]. To circumvent this, several next‐generation strategies are under investigation. Bispecific antibodies utilizing ‘molecular Trojan horses’ (e.g., targeting transferrin or insulin receptors) aim to exploit receptor‐mediated transcytosis to enhance CNS delivery. Additionally, focused ultrasound‐assisted delivery and intranasal administration routes offer promising alternatives to conventional intravenous infusion [147]. Without addressing this penetrance‐efficacy gap, even the most potent antibodies may continue to fail in late‐stage clinical trials.

In summary, the persistent failures in PD immunotherapy trials represent a multi‐factorial challenge encompassing suboptimal target selection, delayed intervention windows, and the insurmountable bottleneck of the BBB. Current evidence suggests that neutralizing extracellular α‐syn via passive immunization alone may be insufficient to halt neurodegeneration. Future breakthroughs will likely hinge not only on refined epitope selection—shifting toward pathogenic oligomers or C‐terminal fragments—but also on the successful integration of advanced delivery technologies that can substantially elevate CNS bioavailability [148].

### 5.4 Meta‐Synthesis of Therapeutic Patterns and Trial Failures

Although extensive clinical development efforts have been made for the activity of α‐syn (such as PD01A, UB‐312) and passive (such as prasinezumab, simanemba) immunotherapies, most trials have failed to achieve their primary clinical endpoints. Firstly, the “time mismatch” phenomenon is prevalent in failed trials. Most interventions were initiated after significant development of neurodegenerative lesions, which limited the possibility of disease improvement. This indicates that immunotherapies may be more effective in the early or pre‐diagnosed stages of PD, but not in the diagnosed disease stage. Secondly, the binding of the target does not necessarily mean clinical benefits. Although many drugs have successfully reduced the levels of α‐syn in the periphery or cerebrospinal fluid, this biochemical effect is not associated with improvements in movement or functional outcomes. This disconnection highlights the incomplete understanding of the causal relationship between extracellular α‐syn clearance and neurodegenerative lesions. Thirdly, the common limitations of current antibodies lie in their emphasis on the periphery. Active immunotherapies and passive immunotherapies mainly target extracellular α‐amyloid protein aggregates, while increasing evidence suggests that intracellular oligomeric substances play a core pathogenic role in the disease progression. Due to the inability of antibodies to effectively penetrate the neuronal cell membrane, this constitutes a critical treatment gap. Fourthly, the insufficient exposure of the central nervous system due to the blood–brain barrier remains a fundamental bottleneck. The brain penetration rate of most monoclonal antibodies is extremely low (less than 0.1% of plasma concentration), which may lead to treatment concentrations at the lesion site being lower than the therapeutic level. Finally, the inconsistency in trial design has led to inconsistent results. Differences in patient selection (early PD patients vs. moderate PD patients), endpoint indicators (clinical scales vs. biomarkers), and trial duration have made cross‐study comparisons complex and may mask potential subgroup‐specific benefits. In summary, these findings indicate that future success in PD immunotherapy will require a shift from single‐target extracellular clearance to multi‐dimensional strategies, including early intervention, intracellular targeting methods, improved central nervous system delivery technologies, and combination therapies.

## 6 Progress in the Pharmacological Treatment of PD

### 6.1 PD Symptomatic Treatment Drugs

At present, the clinical drugs utilized for the symptomatic treatment of PD comprise compound levodopa, anticholinergics, amantadine, dopamine agonists, MAO‐B inhibitors, adenosine A2A receptor agonists, etc.

In the pathological mechanism of PD, neuroinflammation and immune abnormalities are key drivers of disease progression. Currently, commonly used PD treatments, such as levodopa and dopamine agonists, not only improve motor symptoms resulting from dopaminergic neuron loss but also modulate immune responses through multiple pathways. However, their effects depend on drug type, concentration, administration method, and immune cell subtype, often exhibiting “bidirectional regulation” or “concentration‐dependent regulation.” Levodopa exerts antioxidant and anti‐apoptotic effects, regulates T‐cell differentiation in the periphery, and modulates microglial activity in the CNS. Dopamine agonists, by activating D2‐like receptors, can inhibit proinflammatory pathways, regulate immune cell differentiation, enhance anti‐inflammatory effects, and reduce some of the adverse effects associated with levodopa [149].

Levodopa reduces fluctuations and movement complications by transdermal administration compared to oral tablets, providing a noninvasive natural alteration that may help older patients experiencing symptoms of dementia and dysphagia [150]. ND0612 represents a continuous subcutaneous levodopa/carbidopa delivery system in the process of development and is generally safe and well‐tolerated. Ongoing studies (NCT02726386 and NCT04006210) using higher doses as alternatives to gastrointestinal L‐dopa infusions are unknown [151]. CVT‐301, an orally inhaled levodopa powder, was supported by findings that provided resolvable intermittences without increasing dyskinesia and had an acceptable safety profile [152]. The AccordionPill capsule (AP) is under development, and a phase 3 study is underway [153]. Anticholinergic drugs are prevalently employed to tackle tremors and defer the demand for levodopa therapy in young PD patients, and they are also adopted to reduce the dosage of levodopa in patients with advanced disease [154]. Amantadine, as an NMDA receptor antagonist, can effectively alleviate the symptoms of chorea in PD patients who receive levodopa treatment, without exacerbating Parkinson's syndrome [155]. Studies of amantadine as a monotherapy for PD are limited by a heterogeneous patient population [156].

Dopamine agonists (DA) are a class of drugs that can directly activate dopamine receptors and simulate the function of endogenous dopamine. These drugs are often used in patients with PD, especially in the early stages of the disease when patients begin to experience movement disorders caused by levodopa [157, 158]. Monoamine oxidase‐B (MAO‐B) inhibitors can penetrate the BBB and significantly improve patients' motor and nonmotor symptoms, reduce the duration of the “off” phase, and may have neuroprotective effects [159, 160].

### 6.2 Investigational Drugs

In the field of GLP‐1 receptor agonists (GLP‐1R agonists), research on PD has primarily focused on exenatide and liraglutide [161]. The results of an ongoing phase III study of exenatide in mild to moderate patients with PD are undetermined and will be reported in Q3 2024 [162]. In the phase 2 trial related to the effect of liexenatide on the progression of motor deficits in patients with early PD, the trials were restricted, and more extensive and prolonged trials are indispensable to determine the efficacy and safety of this drug in the treatment of PD [163]. Nilotinib might enhance dopamine metabolism and be competent to treat the motor and nonmotor symptoms of PD. A phase II study manifested that nilotinib had a reasonable safety profile, was well tolerated, and could be detected in the CSF of PD patients [164]. The iron chelator, PBT434, is a novel compound of quinazolidinone. The short‐term administration of deferiprone in PD patients is safe and is correlated with the reduction of iron in specific brain regions. These studies provide the basis for future long‐term clinical trials to further evaluate the neuroprotective effects of deferiprone in PD [165, 166]. Ursodeoxycholic acid has been used in the treatment of liver diseases for over 30 years, and the drug is able to promote the production of ATP in mitochondria. Studies have shown that ursodeoxycholic acid may improve the pathophysiology of PD by ameliorating mitochondrial dysfunction [167].

## 7 Conclusions and Future Directions

Experimental evidence highlights a significant association between PD and immune dysfunction. Studies have shown that this association is primarily manifested in several aspects: weakened cellular and humoral immune responses, activation of inflammatory cells, and overall disruption of immune function. Specifically, the immune system of PD patients exhibits obvious abnormalities, such as dysfunction of T cells and B cells, leading to a decline in the body's ability to protect the nervous system. At the same time, inflammatory cells such as microglia and astrocytes are activated, releasing various proinflammatory factors, further exacerbating the process of neuroinflammation. These phenomena independently prove the important role of neuroinflammation in the pathophysiology of PD. Neuroinflammation not only affects the survival of dopaminergic neurons but also may accelerate the progression of neurodegenerative disease through a series of complex molecular mechanisms. For example, chronic inflammation leads to increased oxidative stress, mitochondrial dysfunction, and ultimately neuron death. Additionally, inflammatory responses may disrupt the integrity of the BBB, allowing peripheral immune cells to more easily enter the CNS, thereby aggravating the condition. However, despite the extensive research that has revealed the connection between PD and the immune system, the specific pathogenic mechanisms still need to be further explored. Many key questions have yet to be answered, and the answers to these questions are crucial for understanding the pathogenesis of PD. Furthermore, the temporal relationship between innate immunity and adaptive immune responses and neurodegenerative diseases has not yet been fully elucidated. Current research suggests that innate immune responses may play a critical role in the early stages of the disease, whereas adaptive immune responses may gradually take the lead in the progression of the disease. However, the specific temporal relationships and the underlying molecular mechanisms remain to be further studied.

In summary, although the existing evidence has shown the importance of the immune system in the pathogenesis of PD, a comprehensive understanding of this complex relationship requires more basic research and clinical trials to uncover its secrets.

Many key scientific questions remain unresolved, which continue to limit breakthroughs in immunotherapy. These include the temporal coordination between innate immunity and adaptive immunity, distinguishing causality from correlation in immune abnormalities, and understanding the immune heterogeneity of PD subtypes.

Despite significant strides in decoding the immunopathology of PD, several formidable hurdles continue to impede the translation of immunotherapeutic strategies into meaningful clinical outcomes. First, there is an exigent need for validated biomarkers to enable precise patient stratification and early intervention. Most current clinical trials enroll participants at symptomatic stages when neurodegeneration is already extensive. Identifying robust biomarkers—such as CSF a‐syn seed species, peripheral immune signatures, or neuroinflammatory imaging ligands—is paramount for selecting cohorts most likely to benefit and for defining the optimal therapeutic window. Second, the timing of intervention remains a critical determinant of success. Mounting evidence suggests that immunotherapies targeting a‐syn or neuroinflammation are likely most efficacious during the prodromal or nascent stages of PD. Consequently, future research must prioritize longitudinal designs within early‐diagnostic cohorts to evaluate true disease‐modifying potential. Given the multifactorial nature of PD, the limitations of monotherapy directed at a single pathway (e.g., extracellular a‐syn clearance) are becoming increasingly apparent. Rational combination therapies—incorporating immunomodulation with anti‐inflammatory agents, mitochondrial stabilizers, or gene therapies—may yield synergistic effects and overcome pathway redundancy. Furthermore, surmounting the limitations of CNS drug delivery remains a core challenge. Innovative approaches, such as bispecific antibodies engineered for receptor‐mediated transcytosis, focused ultrasound‐assisted BBB modulation, and intranasal delivery systems, offer promising avenues to enhance cerebral drug exposure and therapeutic potency. Future research must also integrate a precision medicine framework. In light of the profound heterogeneity in PD—encompassing immune profiles, genetic backgrounds, and progression rates—tailoring personalized strategies based on immunophenotyping and molecular subtyping could significantly amplify therapeutic impact.

Finally, the field should pivot away from the conventional paradigm of purely extracellular a‐syn neutralization toward a more holistic mechanistic understanding. This involves targeting intracellular pathogenic species, modulating neuroimmune cross‐talk, and elucidating the dynamic interplay between innate and adaptive immunity across different disease stages. Collectively, these priorities underscore a necessary shift from empirical, monolithic approaches toward early, targeted, and multimodal immunotherapeutic strategies, which may ultimately unlock the full potential of immune‐based interventions in PD.

## Author Contributions

Y.P. received funding support and developed the research hypothesis. Y.P., X.‐h.K., S.‐y.Y., X.Z., S.K., J.L., M.‐q.D., L.‐x.L., D.‐y.J., Q.C., H.J. wrote the main manuscript. The final manuscript is the end product of the joint writing efforts of all authors.

## Funding

This research was funded by the Scientific Research Project of the Hunan Provincial Health Commission, People's Republic of China (Grant No. C202303076574 to YP), Key Plans of Hunan Administration Traditional Chinese Medicine, PR China (No. Grant No. 2023039 to YP), the University‐Hospital Joint Fund of Hunan University of Chinese Medicine, PR China (Grant No. 2022XYLH198 to YP), the Fund for Creative Research Groups at the Affiliated First Hospital of Hunan Traditional Chinese Medical College, PR China (Grant No. 2021B‐003 to YP), Fund for Research Chief of Clinical Department of Affiliated First Hospital of Hunan Traditional Chinese Medical College, PR China (to YP), and the Technology Plan Project of Zhuzhou City, Hunan Province, PR China (Grant No. 2021‐009 to YP).

## Ethics Statement

The authors have nothing to report.

## Consent

The authors have nothing to report.

## Conflicts of Interest

The authors declare no conflicts of interest.

## Data Availability

The data that support the findings of this study are available from the corresponding author upon reasonable request.
